# Algorithmic management and human-centered task design: a conceptual synthesis from the perspective of action regulation and sociomaterial systems theory

**DOI:** 10.3389/frai.2024.1441497

**Published:** 2024-09-25

**Authors:** Carsten Röttgen, Britta Herbig, Tobias Weinmann, Andreas Müller

**Affiliations:** ^1^Institute of Psychology, Work and Organizational Psychology, University of Duisburg-Essen, Essen, Germany; ^2^Institute and Clinic for Occupational, Social and Environmental Medicine, LMU University Hospital, LMU Munich, Munich, Germany

**Keywords:** digitalization, artificial intelligence, work design, Job Demands-Resources Model, work stress, motivation, self-determination

## Abstract

This paper aims to explain potential psychological effects of algorithmic management (AM) on human-centered task design and with that also workers’ mental well-being. For this, we link research on algorithmic management (AM) with Sociomaterial System Theory and Action Regulation Theory (ART). Our main assumption is that psychological effects of sociomaterial systems, such as AM, can be explained by their impact on human action. From the synthesis of the theories, mixed effects on human-centered task design can be derived: It can be expected that AM contributes to fewer action regulation opportunities (i.e., job resources like job autonomy, transparency, predictability), and to lower intellectual demands (i.e., challenge demands like task complexity, problem solving). Moreover, it can be concluded that AM is related with more regulation problems (i.e., hindrance demands like overtaxing regulations) but also fewer regulation problems (like regulation obstacles, uncertainty). Based on these considerations and in line with the majority of current research, it can be assumed that the use of AM is indirectly associated with higher risks to workers’ mental well-being. However, we also identify potential positive effects of AM as some stressful and demotivating obstacles at work are often mitigated. Based on these considerations, the main question of future research is not whether AM is good or bad for workers, but rather *how* work under AM can be designed to be humane. Our proposed model can guide and support researchers and practitioners in improving the understanding of the next generation of AM systems.

## 1 Introduction

Intelligent technological systems that have the capability to learn and to make autonomous decisions through algorithmic pattern detection permeate and fundamentally transform our work life (Cascio and Montealegre, 2016). Such systems are also increasingly used to take over decision-making in organizations from human actors such as managers (Benlian et al., 2022). These types of technological systems that overtake managerial decisions at work are referred to as data-driven or algorithmic management systems (AM) (Lee et al., 2015). So far, AM are mainly used to manage work in the so-called platform economy (e.g., Uber, Amazon MTurk) (Rosenblat and Stark, 2016), or in warehouse logistics (Schmierl et al., 2022) with rather low skilled jobs. However, AM is also beginning to transform traditional organizations and higher qualified jobs, such as engineering (Bakewell et al., 2018) or healthcare (Mashar et al., 2023). Consequently, one can predict that AM will be an important factor for the design of future workplaces which deserves a broad attention of I-O psychology concerned with humane work design.

The increasing shift in agency from people to technology by the introduction of AM at work is seen as a fundamental new quality of work (Benlian et al., 2022; Gagné et al., 2022; Kellogg et al., 2020; Parker and Grote, 2022). For example, AM is considered to radically reconfigure the “contested terrain” of organizational control as one of the most fundamental aspects of the employer – employee relation (Kellogg et al., 2020). As the ability to influence one’s own work is one of the key job resources for workers, AM is also expected to impact the mental well-being in future work places (Kinowska and Sienkiewicz, 2022).

Although comprehensive I-O psychological research in the last decades has expanded our understanding on the design of a meaningful and healthy work environment (e.g., Parker et al., 2017a), the psychological understanding of AM and its possible consequences for workers’ well-being is only just beginning. With this conceptual paper we build on empirical and descriptive studies of AM practices to complement and extent recent psychological knowledge about the impact of AM on work design (Parent-Rocheleau and Parker, 2022) and workers motivation (Gagné et al., 2022) by looking at AM from action regulation theory (ART) (Frese and Zapf, 1994; Hacker, 2003; Zacher, 2017), and sociomaterial-system perspective (Cascio and Montealegre, 2016; Landers and Marin, 2021; Orlikowski and Scott, 2008). We believe that by integrating both theoretical perspectives new I-O psychological insights about the human centered design of algorithmically managed workplaces can be derived:

(1) So far, the starting point for the consideration of AM has mainly been its managerial functions. The perspective of ART will additionally contribute to identify psychologically important functions of AM from the perspective of involved *workers.* ART will help to further reveal the underlying mechanisms through which the functions of AM will affect the design of work tasks, work behavior, and finally also workers’ well-being and vice versa. Thus, the perspective of ART allows to further systematize the psychological understanding of AM functions across a wide range of jobs and industries. (2) I-O psychological research on work design has often been criticized to neglect that psychological and social phenomena in organizations are related with material aspects of technologies (Landers and Marin, 2021; Orlikowski and Scott, 2008; Parker et al., 2017b). The sociomaterial-system perspective highlights that AM is a fusion of material aspects (e.g., computer networks, mobile digital devices, or the interface of a software program) as well as psychological/social aspects (e.g., goals and values of involved organizations and workers) (Orlikowski and Scott, 2008). Taking the sociomaterial-system perspective will therefore contribute to deepen our theoretical understanding about which “objectifiable” characteristics and functions of AM affect workers’ actions and well-being. (3) From a practical perspective, this understanding of the interrelation between the material and psychological/social aspects of AM is one important precondition to name and substantiate concrete starting points for the human-centered design of modern digitalized workplaces. Thus, our approach helps to better understand the effect of technology on humane work design, an issue that is rarely the focus in existing I-O research so far (Parker et al., 2017b).

In the following sections, we will first describe what organizational research to date understands by AM. We then give a brief overview of the main assumptions of ART as well as of the conception of technologies as sociomaterial configurations. In a next step, we develop a new theoretical founded definition of AM before finally deriving propositions about the effects of AM on human-centered work design.

### 1.1 Algorithmic management

The term AM refers to learning algorithms that carry out automated data-driven coordination and control of workers without explicit involvement of human managers or other human agents at work (Benlian et al., 2022; Gagné et al., 2022; Möhlmann et al., 2021; Noponen et al., 2023; Parent-Rocheleau and Parker, 2022). AM *takes over typical functions and responsibilities of lower and middle managers* like assigning tasks, scheduling work, monitoring task accomplishment, evaluating workers performance, providing rewards or sanctioning, and even making human resource management decisions, like termination of work (Benlian et al., 2022; Gagné et al., 2022; Parent-Rocheleau and Parker, 2022).

The implementation of AM is tied to interconnected digital technologies that enable the collection, storage, processing, and transmission of data: Basically, learning algorithms are informed by large quantities of data that are continuously recorded by digital devices (e.g., cell phones, tablets, or handhelds) of involved stakeholders like workers and customers. Depending on the job, a multiplicity of data of varying depth and magnitude is recorded, such as behavioral data of workers (e.g., movement data), their active inputs (e.g., about completed tasks), but also physiological reactions (e.g., galvanic skin response) (Cram and Wiener, 2020). The data are transferred via internet connections and stored on digital platforms where they are processed, and from where algorithmically generated instructions and feedback are sent back to the digital devices of workers.

From an economic perspective, AM particularly offers enormous growth opportunities and adaptability of business models, by facilitating the flexible on-time coordination of large numbers of on-demand tasks, like food delivery or transport of passengers (Benlian et al., 2022; Möhlmann et al., 2021). One prototypical example is the globally operating platform company Uber that operates a smartphone app which connects mainly independently operating drivers with passengers to provide “ridesharing” services (Rosenblat and Stark, 2016). Uber is an impressive example that AM can operate global companies that have a shareholder value comparable to large traditional corporations, such as Volkswagen, with a comparatively small amount of material resources and regularly employed workers. With the development of this so called “gig economy,” AM has opened a whole new labor market providing the opportunity to earn money with a very low entry threshold (Wu and Huang, 2024).

From the perspective of workers, these potentially tremendous entrepreneurial advantages seem to be outweighed by an array of drawbacks. Recent initial reviews indicate that current applications of AM have predominantly negative effects on the quality of work (Parent-Rocheleau and Parker, 2022), as well as on individual outcomes such as motivation (Gagné et al., 2022) and other aspects of mental well-being (Kinowska and Sienkiewicz, 2022). For example, similar to traditional efficiency-driven Taylorist management systems, it is suspected that AM might be related to higher workload and reduced job autonomy (Kellogg et al., 2020; Parent-Rocheleau and Parker, 2022); a combination that is well-known to be a significant psychosocial health risk for workers (Karasek, 1979; see also Theorell et al., 2015). Even more significant, AM is also suspected to possess entirely new qualities compared to traditional management approaches particularly by replacing important social agents at work like human managers, thus literally contributing to a “dehumanization” (Lamers et al., 2022) of work, and by making work more opaque and unpredictable compared to repetitive but predictable traditional work systems (Noponen et al., 2023).

However, AM can be designed and implemented in quite different ways, so that the psychosocial effects of AM are not necessarily predetermined (Benlian et al., 2022; Cram and Wiener, 2020; Noponen et al., 2023). One of the few available simulation studies indicates, that single aspects of AM can also be designed in a supportive and motivating way if psychological needs of workers are taken into account (Sailer et al., 2017). From a psychological perspective, it is therefore important to better understand the opportunities for and limitations of the human-centered task design under the conditions of AM.

### 1.2 Action regulation theory

Human action like taking care of a person, delivering a good, repairing a car is (still) the core of work (Hacker, 2003). Action Regulation Theory (ART; Frese and Zapf, 1994; Hacker, 2003) captures the cognitive processes of human action regulation and explains its relation to desirable individual outcomes, such as workers’ well-being, personal growth, intrinsic motivation, and good performance. ART assumes that these desirable individual outcomes are closely intertwined with the extent to which the work environment either promotes or impedes *autonomous* actions of workers with scope for decision making and high levels of personal control. With that, ART ties together task characteristics, like job autonomy, with psychological processes and states, like motivation and well-being. Therefore, ART can be useful to deepen our psychological comprehension of AM as part of the work environment, and to better understand the potential psychological effects of AM.

Basically, ART illuminates human action from two perspectives (Frese and Zapf, 1994; Hacker, 2003): On the one hand, the theory considers action as cyclical *sequences* of action phases with a certain logical order: goal development, planning and orientation, executing and monitoring, and feedback. In addition, the theory distinguishes three *hierarchical* organized cognitive levels of action regulation (Hacker, 2003): On the sensumotor level actions are automated and regulated without conscious attention (e.g., riding a bicycle). On the knowledge-based level well-practiced actions are executed that are based on plans stored in memory and that must be adapted to a specific situation. Actions on that level can but do not have to be consciously regulated (e.g., navigating the bicycle on the well-known route to work). Action regulation on the highest, the intellectual level, is characterized by a conscious development and activation of goals and plans for the regulation of complex activities. This takes place when no ready-to-use pattern of activity exists, that is, when non-routine actions are regulated (e.g., finding a way in an unknown town).

ART particularly emphasizes the importance of goals, in the sense of the mental representation of a future outcome, for human action (Hacker, 2003). Goals trigger actions, direct attention during action, and are the benchmarks to evaluate the progress of action. Thus, goals align the complete action sequence. Goals also integrate cognitive as well as motivational processes of action regulation (Frese and Zapf, 1994): From a cognitive perspective, the iterative self-regulated development of adequate goals is for example an integral demand of intellectual level action regulation in non-routine problem-solving tasks (Hacker, 2003). From a motivational perspective, specific and difficult goals increase the perseverance and effort of workers during task accomplishment (Locke and Latham, 2016). Moreover, self-set and internalized goals contribute to self-determined and intrinsically motivating work (Deci et al., 2017).

From these basic assumptions ART derives normative standards for the humane design of work tasks, that are in accordance with well-known theories about health-related work design, like the Job Demands-Resources Model (JD-R, Bakker and Demerouti, 2017), that distinguishes between job demands, i.e., working conditions that require physical and mental energy from the employee and are mainly related to strain processes, and job resources, i.e., working conditions that directly or indirectly satisfy basic human needs and might therefore trigger motivational and salutogenetic processes. Basically, the JD-R model (Bakker and Demerouti, 2017; Bakker et al., 2023; Demerouti, 2020) distinguishes between two kinds of working conditions that are related with two kinds of health related psychological mechanisms: (a) Job demands require the use of physical and mental energy. Mediated through stress-related mechanisms, they can represent risk factors for mental health. Health risks exist, for example, when job demands are experienced as uncontrollable (Karasek, 1979) or when they hinder or complicate the accomplishment of work tasks (LePine et al., 2005). (b) Job resources can strengthen mental health through motivational mechanisms. In this context, job resources are those working conditions that directly correspond to basic human needs or that are instrumental in satisfying these basic needs [e.g., latitudes at work that correspond to the basic need for autonomy and self-determination (Deci et al., 2017; Hackman and Oldham, 1976)]. Job resources can also mitigate the potentially negative effects of work demands (Karasek, 1979). Accordingly, health risks arise when job resources are not available to a sufficient extent (Lesener et al., 2019). Due to its good empirical evidence (Lesener et al., 2019), its high degree of generalization and its compatibility with other central psychological theories of work design (Hacker, 2003; Hackman and Oldham, 1976; Karasek, 1979), the JD-R model is suitable to explore and classify potentially novel conditions of digitalized work (Demerouti, 2020).

#### 1.2.1 Job resources from the perspective of ART

From the perspective of job resources, which in ART are referred to as regulation opportunities, ART suggests the concept of *complete tasks* and activities as a gold standard for work design (Hacker, 2003): Work tasks are sequentially complete when their design offers latitude about the action sequence described above, particularly having the opportunity to develop self-set goals as well as to plan the action steps and measures to reach these goals. Moreover, tasks are hierarchically complete when they require all levels of action regulation, i.e., automated sensumotor regulated actions, as well as knowledge-based and intellectual regulated actions. A typical example of an incomplete task in a “traditional” job would be a partialized routine task on an assembly line in the automobile production, where a narrowly defined action step (such as attaching a car body part) simply must be performed over and over again, without requiring any specific goal development or planning. A more complete task would be the automobile production by semi-autonomous groups that can co-determine and plan their own work processes, as was found in some Volvo plants until the 1990s (Sandberg, 1993).

Complete tasks contain specific job resources that are well-known from other established work design-models like the Job Demand-Control Model (Karasek, 1979) and the Job Characteristics Model (Hackman and Oldham, 1976; Humphrey et al., 2007): The main characteristic of complete tasks is a high level of *job autonomy* that offers the worker leeway to develop goals and exert control about the complete sequence of action (Frese and Zapf, 1994; Hacker, 2003). Complete tasks should also be related with further intrinsically motivating task characteristics such as *task identity*, i.e., employees’ perception that they contribute to a complete piece of work (Hackman and Oldham, 1976; Humphrey et al., 2007). Another important job resource included in complete tasks is *feedback*, that provides workers with helpful information about their progress toward reaching goals and thus with learning opportunities (Zacher, 2017).

According to the Job Demand-Control Model (Karasek, 1979), complete tasks should therefore help to avoid chronic stress and associated health risks because particularly the higher job autonomy or decision latitude increases the internal control of workers to adjust their work tasks according to their personal abilities and skills (Hacker, 2003). Moreover, according to the Job Characteristics Model (Hackman and Oldham, 1976; Humphrey et al., 2007) the perception of job autonomy, task identity and helpful feedback should increase workers’ perceptions of psychological states like responsibility, meaningfulness, and accomplishment in their work, which altogether should contribute to intrinsic motivation.

ART also suggests that *transparency* and *predictability* of work tasks are further important job resources that enable workers to take control over work processes (Frese and Zapf, 1994). They both somewhat go beyond the characteristics of the specific work task and also relate to the design of the work organization and the wider work environment. Transparency refers to the knowledge about the meaning of relevant task related information (e.g., the meaning of a specific work object, like a tool or a working material) and allows the worker to develop an adequate operative image system, i.e., an adequate mental model, of its work task (Hacker, 2003). A lack of transparency makes it difficult to interpret information appropriately and limits the possibilities to develop adequate task goals and action plans. Whereas transparency refers to the present work situation, predictability refers to the possibility to foresee future work tasks, changes, or problems. Predictability is an essential prerequisite for forward-looking action planning that goes beyond a mere reaction to action stimuli and has been shown to be a further important health relevant job resource for employees (Väänänen et al., 2008).

#### 1.2.2 Job demands from the perspective of ART

In ART, *job demands* are conceptualized as regulation requirements that are related to properties of the hierarchic-sequential organization of action (Frese and Zapf, 1994). In accordance with the Challenge-Hindrance Stressor Framework (LePine et al., 2005), regulation requirements are primarily seen as motivating and learning-promoting intellectual demands (i.e., challenge demands). On the one hand, coping with these intellectual demands requires individual mental and physical resources. These individual resources will be exhausted at some point and must therefore be regenerated in order to avoid impairment of well-being (Sonnentag et al., 2022). On the other hand, they are motivating, as the accomplishment of challenging demands meets our basic need for competence (Deci et al., 2017). Human-centered work should therefore not aim to reduce such challenging task demands, but to design demands in such a way that they correspond to the skill level of the employees.

Complete tasks not only contain heath promoting and motivating job resources, but they also require a higher level of cognitive demands, as goals and action plans may need to be developed and adapted during action execution. We want to distinguish between two types of challenging job demands that go along with complete tasks: task complexity and problem solving that are particularly important from the perspective of ART.

Whereas job control can be defined in terms of available decision possibilities, *task complexity* implies decision necessities. Task complexity increases with the number of task goals and the degree of interconnection between these goals. Higher task complexity leads to a higher degree of regulation requirements, as pursuing one task goal positively or negatively affects multiple other task goals. Previous research suggests that high task complexity promotes satisfaction, as coping with complex demands goes hand in hand with a high sense of competence; however, it might also be related with the experience of overload, as employees might perceive those tasks as too complicated and overtaxing (Humphrey et al., 2007).

*Problem solving* particularly focuses on the extent to which a task requires the development of novel solutions or ideas (Humphrey et al., 2007). Particularly, tasks that are regulated on the intellectual level are often novel tasks with initially unclear goals and therefore uncertain action plans. While this implies high information processing demands, it also provides the potential to learn and grow by solving unknown problems (Frese and Zapf, 1994).

In line with the Challenge-Hindrance Stressor Framework (LePine et al., 2005), ART also enables the derivation of a taxonomy of stressful and demotivating hindrance demands, in terms of action *regulation problems*. Roughly, regulation problems can be distinguished in *regulation obstacles*, *regulation uncertainty*, and *overtaxing regulation* (Frese and Zapf, 1994): Regulation obstacles refer to external barriers or hindrances (e.g., unexpected disruptions) that individuals encounter when trying to accomplish tasks or goals. Regulation obstacles unnecessarily increase the effort required to perform a task and might contribute to frustration and stress experience of workers (Baethge et al., 2015). Regulation uncertainty refers to the ambiguity or lack of clarity in one’s task goals, or action plans. Uncertainty can stem from factors such as unclear instructions, conflicting goals, or rapidly changing circumstances, and lead to insecurity and doubts about the clear path forward to goal accomplishment. Finally, overtaxing regulation occurs when a work task places excessive demands (e.g., tight deadlines, simultaneous tasks, information overload) on the workers. Tasks can therefore be experienced as overwhelming and unmanageable (Frese and Zapf, 1994; Zacher and Frese, 2018).

With the introduction of AM, parts of the action sequence are transferred from humans to algorithms (Parent-Rocheleau and Parker, 2022) changing the job demands and resources for workers and with that also the design of their work tasks.

In sum, from the perspective of ART, the psychological assessment of AM should therefore consider the extent to which AM influences the design of work tasks—in particular regulation opportunities (i.e., job resources), motivating and learning-promoting intellectual demands (i.e., challenge demands) or stressful regulation problems (i.e., hindrance demands).

### 1.3 Technologies as sociomaterial systems

As stated above, AM is tied to technologies. Landers and Marin (2021) define technologies as “[…] a collection of enduring physical and/or digital materials that dynamically afford individual and/or collective goal-directed action” (p. 240). Thus, one basic purpose of a technology is the reinforcement and enhancement of human capabilities to reach goals.

This definition shows the close link between technology and action regulation. For example, the physical and material features of a hammer—its sturdy long handle and heavy head—enhances the transfer of our arm power to or exert physical forces on objects. Without such tools, with our bare hands or minds, even rather simple tasks, like driving a nail into a wall to hang up a picture, would be very difficult or even impossible to perform. Similarly, the databases, internet connections, digital devices, and software designs of an AM system enable a worldwide expansion of business models of platform companies like Uber that would not be possible without such digital technologies. A technology is therefore seen as a merger of individual/social aspects—e.g., individual or collective goals as well as the human knowledge and capabilities to reach these goals—and material aspects—e.g., the compilation of physical or digitalized features of a tool, machine or computer—that are specifically designed to enhance human capabilities for goal-directed behavior. Technologies thus are often seen as *sociomaterial configurations* (Leonardi, 2012), i.e., a functional amalgamation of material and human/social aspects.

In this regards, several authors (Landers and Marin, 2021; Orlikowski and Scott, 2008; Zammuto et al., 2007) refer to Gibson (1977) concept of *affordances* that gets to the essence of the sociomaterial configurations of technologies and interrelate them with psychosocial phenomena and processes. “Affordances can be defined as the perception of whether the features of a technology can be used to achieve goal-directed actions” (Landers and Marin, 2021, p. 240). As such, affordances are possibilities and at the same time restrictions for action that arise from the connection between people and material objects (Gibson, 1977).

The perspective of affordances helps to understand how the objective design of our work environment facilitates or impedes or even precludes human actions. For example, a hammer affords actions in which physical forces must be exerted on other objects. At the same time the nature of a hammer impedes other actions. It is less suitable for cutting materials precisely or it will hardly be ever used to paint an object. In the same way, the AM system of Uber is optimized to provide a flexible demand-driven transport service for passengers. But from the drivers’ point of view, this might come at the expense of exerting control about their work (Rosenblat and Stark, 2016). Thus, the affordances of the technical design of our working environment may not be determining, but at least is paving our behavior by enabling or hampering goal-directed actions.

Affordances can be conceptually distinguished from operationalizations of psychosocial task design characteristics such as job autonomy, or task complexity (Humphrey et al., 2007), that usually lack a direct reference to the “material” working environment. The consideration of affordances should therefore provide additional information and concrete conclusions about specific starting points for task design, which are often still missing in current I-O psychological research (Parker et al., 2017b). We assume that affordances of AM affect task design and postulate that ART can explain these effects. In the following two sections, we will bring these perspectives together.

## 2 Definition of algorithmic management from the perspective of action regulation and sociomaterial system theory

From the synthesis of the theoretical perspectives introduced above, it can be derived that the main affordances of AM systems from the perspective of the acting worker are goal-setting, action-planning, scheduling, monitoring, and feedback. These affordances correspond with the functions of AM reported elsewhere (Benlian et al., 2022; Gagné et al., 2022; Parent-Rocheleau and Parker, 2022).

From the action theoretical concept of complete tasks one can further conclude that the more of these functions an AM system incorporates, the more incomplete a task is from the perspective of workers. It is therefore likely that AM has an impact on job resources like job autonomy and job control as well as on the extent of learning-promoting and motivating challenge demands like job complexity as well as on stressful and demotivating hindrance demands like regulation uncertainties.

Moreover, in addition to the individual functions and affordances of AM mentioned above, we assume that the “completeness” of AM is an affordance in itself with an own quality. Because the single functions of AM should, according to ARTs concept of complete tasks, have a close inherent logical relationship, we believe that they also jointly affect work design and mental well-being, and therefore must also be studied together and not solely separately.

Consequently, we suggest the following working definition of AM: *An algorithmic management system is a sociomaterial system which affords the work behavior of employees through rule-based computed (algorithmic) goal-setting, action-planning, scheduling, monitoring, and feedback, without explicit involvement of human managers or other social agents at work. The amount and extent of algorithmic control of these functions indicate the* “*completeness of AM*.”

In the following, we want to derive our propositions on the relationship between the completeness of AM and the quality of work design based on ART and examine them with the available literature.

To illustrate the current knowledge on the effects of AM on the quality of work and action regulation, use cases of work which is already managed by algorithms are presented. The description of these use cases follows the sequence of action steps according to ART. This will shed light on the perspective of workers under AM working conditions. The effects of AM on the quality of task design and thus on workers mental well-being are discussed after the use cases to (1) highlight the key differences between AM work quality and traditionally managed work and (2) to show the similarities of AM work quality in different work contexts.

## 3 Use cases of algorithmic management

### 3.1 Algorithmic management in the ridesharing industry

The advent of algorithmic management has ushered in a new era in the so-called gig economy. The gig economy is a part of the labor market in which workers engage on a job-by-job basis (Manyika et al., 2016). For example, in contrast to traditional taxi services, companies like Uber and Lyft do not employ drivers but provide a platform which enables the matching of self-employed drivers and customers. This puts the drivers into a freelance status, reducing the employer obligations (e.g., occupational health and safety) and shifting a great part of the business risk from companies to workers (Rosenblat and Stark, 2016). To organize this complex network of drivers, algorithmic systems are used (Lee et al., 2015).

Once workers are registered in the Uber app, they are offering transportation of other persons like traditional taxis would do. The interaction between drivers and the algorithmic management system begins with matching customer and driver, where algorithms define targets based on a myriad of variables (Rosenblat and Stark, 2016). Previous performance, customer ratings, and geographical considerations all contribute to a dynamic goal-setting process. Once a customer requests a transportation, the algorithm chooses the closest driver in that area. The driver cannot influence whom he or she should transport or where the journey should go to. In fact, almost no information about the customer is shared with the driver before the ride is accepted. The only way to “decline” a ride is to wait out the 15 s time window in which the ride needs to be accepted (Cropanzano et al., 2023; Lee et al., 2015).

As drivers embark on their journeys, algorithms function as co-pilots, planning actions through a continuous exchange of information. Real-time variables such as passenger demand, traffic conditions, and driver proximity are considered. The algorithmic system plans routes, selects rides, and adapts to unforeseen circumstances. Once a driver picks up a customer, the app provides the routing to the target destination. In addition, the algorithm already starts finding potential new clients for the driver once the current ride is over. In contrast to traditional taxi drivers, ridesharing drivers do not need to know the area they are working in. Even if they knew any shortcuts, the app would provide the directions to take (Wood, 2021).

To ensure effective scheduling, algorithms anticipate and orchestrate driver movements. Leveraging predictive analytics, these systems forecast demand, dynamically allocating drivers to specific locations. Drivers, guided by the anticipatory algorithms, become integral components of a “synchronized dance,” strategically positioned in high-demand areas to meet the ebb and flow of the ridesharing ecosystem (Rosenblat and Stark, 2016).

The driver’s performance is constantly monitored. Metrics such as completion rates and customer ratings are observed in real-time. Deviations from performance standards trigger immediate adjustments, shaping the algorithm’s decision-making process. This interactive monitoring fosters a dependent relationship, as drivers strive to align their actions with the algorithmic expectations to maximize efficiency and earnings (Rosenblat and Stark, 2016).

Feedback to drivers is provided by algorithms through real-time evaluations and performance dashboards. Passengers provide immediate feedback, shaping driver behavior. Simultaneously, performance metrics and earnings insights empower drivers to adapt their strategies (Rosenblat and Stark, 2016).

### 3.2 Algorithmic management in the food delivery industry

Algorithmic management has also revolutionized the food delivery industry, introducing intelligent systems that dynamically guide the actions of delivery drivers (Ivanova et al., 2018). In most food delivery companies the work starts with logging into the respective app. Once online, workers receive requests for delivery and the corresponding restaurant address (Veen et al., 2020). In some apps workers have approximately 10 s to accept a request, in some apps the default option is acceptance with 10 s to decline (Veen et al., 2020).

Once the request is accepted, the work follows a strict step by step process managed by the app. After picking up the order at the restaurant the worker needs to confirm the completeness of the order. Only after this step the delivery address is provided (Ivanova et al., 2018). The app proposes the route to the client. Although the rider is able to choose another route, the app will often nudge the worker with notifications in case the chosen route seems to be slower (Ivanova et al., 2018).

Real-time demand patterns, geographic variations, and driver availability inform the dynamic scheduling process. Drivers are in theory able to choose their shifts according to their preferences and availability through the platform interface. In practice however, workers are often organized in badges depending on their rating by the app. The highest rating badge can choose their work schedule for the next week first, then the second badge and so forth (Ivanova et al., 2018). That way only workers in full compliance with the apps rating criteria have some level of control about their working schedule (Griesbach et al., 2019).

Continuous monitoring is a hallmark of algorithmic management, with real-time data informing performance assessments. Workers are being monitored via GPS and customer rating are gathered (Ivanova et al., 2018).

Automated feedback systems provide drivers with real-time feedback. Although this information is helpful for the worker, it is not directly obtained by the worker through interaction with the customer or restaurant but only received through the app with no means to ask for clarification if needed.

### 3.3 Algorithmic management in warehouses

As an example of more traditional work arrangements that has been fundamentally changed by algorithmic management, warehouse logistics show a substantial integration of AM into the work process. Some in fact argue that human workers are as much integrated into a system as a robot would be (Delfanti, 2019). Although there are still human managers present in warehouses, their interaction with workers is reduced to a minimum; and remaining managers often rely on the algorithmic management system to receive information about the workers productivity (Delfanti, 2019).

All workers receive a barcode scanner, often referred to as “gun” at the beginning of their shift as their main instrument of work. This barcode scanner is the equivalent of the app in the platform economy as it mediates between workers and management, setting goals, assigning, and planning tasks, monitoring the work completion and providing feedback (Delfanti, 2019; Gent, 2018).

The work itself is organized into four core processes: receive incoming wares, stow incoming wares into the storage system, pickup outgoing wares out of the storage system and pack outgoing wares for shipment. At the “receive” workstation, workers unpack pallets and scan the barcodes of the wares and pack them on a tote. The tote travels to the “stow” area, where workers group the wares into bins and carry those to the automated storage system. “Pick”-workers retrieve items from the storage system and bring them to sorting workstations. In the “pack” area, workers receive, pack and label wares for shipment. Work starts once workers pickup their barcode scanner and scan their work batch at the beginning of each shift (Delfanti, 2019).

Goals are set and communicated by the barcode scanner to the worker. Once an order has been placed, the system calculates which worker should retrieve the item from the storage system depending on the locations of the item and the worker (Delfanti, 2019). The goals are based to the data previously collected from workers (Gent, 2018).

In case of batches of orders, the barcode scanner provides not the complete overview, but only the next item to pick up to the worker. That way the sequence of item retrieval is “known” only to the AM system and workers cannot look ahead past their current activity (Delfanti, 2019). At the packing workplace, the scanner tells the packer which box size to use (Gent, 2018).

The time when a worker needs to receive, stow, pick up or pack an item is determined by customer orders and communicated by the barcode scanner to the worker. There is no option for the worker to change the time or sequence of the actions required to fulfill the task (Delfanti, 2019; Gent, 2018).

All information about the work is gathered and communicated by the barcode scanner. It constantly monitors the location of the worker and calculates the speed workers need to fulfill their tasks, such as items packed per hour (Delfanti, 2019; Gent, 2018).

The information about the workers performance is provided to the worker and to the warehouse management. That way performance feedback is available to workers to know if they meet the set targets. The barcode scanner does not provide any information to improve in case these targets are not met, however. It is not even communicated what data the performance evaluation, communicated in percent target achievement, is based on. Even warehouse managers have no oversight therefore fully dependent on that system (Delfanti, 2019; Gent, 2018). The inherent necessity of chaotic warehousing not only affects workers and managers but also leads to a complete dependence of the company/organization on the system as all overview and control of locations and stock is transferred to the technical system.

## 4 Propositions on the effects of algorithmic management on human-centered task design

From an ART perspective, one can conclude out of these three use cases that AM is associated with sequentially and hierarchically incomplete work tasks, which in turn might have an impact on task design and indirectly also on the stress experience and motivation of workers:

1. Under AM workers usually cannot autonomously decide about their task goals. The *setting of goals* is done by the algorithm and goals are merely communicated to the worker via technical devices. Within flexible gig work, workers are additionally often motivated by nudges or additional extrinsic incentives to pursue these goals. For example, surge pricing is used to increase ridesharing driver availability in certain areas or to a certain time of the day with many customers (Rosenblat and Stark, 2016). Moreover, task goals are strongly connected to real time performance data and customer feedback which can result in dynamic changes of goals and limited predictability (Griesbach et al., 2019; Lee et al., 2015; Rosenblat and Stark, 2016). Particularly, the latter distinguishes AM from traditional efficiency-oriented forms of work organization, such as externally determined but predictable assembly line work, and it can be assumed that this severely restricts the workers’ control over the entire task process.
2. Also the *planning* of required actions is mostly done by the algorithm (e.g., negotiating prices, identifying the fastest route to the destination, selecting the next product to pick from a shelf) (Gent, 2018; Wood, 2021).
3. The same applies to *scheduling*. For example, the Uber algorithm is said to be optimized for matching drivers and customers to provide the drivers with as many customers as possible and the customers with as little waiting time as possible. This is accomplished by using real time data provided by drivers and customers. Hereby, the amount of processed data considered by the algorithm is larger than humans can process. At the moment, in most cases, this data is not made available to the drivers, nor is it processed in a way that would be completely understandable for the drivers. Thus the drivers can often only choose when to start working and when to stop (Lee et al., 2015). The same is true for the food delivery services (Ivanova et al., 2018). The chaotic storage systems in warehouses have a similar effect. Workers can hardly know where a certain product is stored, so only the algorithm can determine where and when to pick up which product to ensure an efficient workflow (Delfanti, 2019). Thus, action planning and scheduling by AM may not only reduce the challenging intellectual regulation requirements for workers. It might also limit the transparency of their work task and conditions for task execution, which are necessary to get an adequate mental model or, in action regulation terms, an *operative image system* of the work task. Both are a prerequisite for goal setting and action planning and thus individual control over the work process (Hacker, 2003).
4. In a similar vein is the *monitoring* of actions performed mainly by technical devices (Griesbach et al., 2019; Rosenblat and Stark, 2016). Monitoring in AM tends to be mainly used to evaluate the performance of workers, which seems to have an additional effect on workers focus on their actions. As for example the rating by customers is an important factor for future earnings, Uber drivers tend to optimize their relationship with the customers at their own expense to receive better ratings (e.g., providing free drinks) (Rosenblat and Stark, 2016). Thus, workers under AM tend to “work for data,” which means that they focus their action regulation more on the aspects of a task that are recorded by the monitoring system and less on the things that seem relevant, meaningful and motivating to them (Moore and Robinson, 2016). Moreover, monitoring might increase the quantitative demands of workers. For example, warehouses worker, where the performance monitoring is more focused on the number of simple tasks completed in a given timeframe, workers are forced to increase their work speed (Gent, 2018).
5. Lastly, also the *feedback* is provided by a technical device. AM usually provides workers with timely and understandable feedback, e.g., whether a task has been finished or how many tasks have been completed. This information may strengthen the feeling of workers’ mastery and their experience of competence (Gagné et al., 2022). However, certain aspects of feedback might not always be acceptable to the worker (Delfanti, 2019). This again might be due to the perceived lack of legitimacy of the feedback and the missing transparency of the feedback criteria, because studies report that some sources of information are not completely reliable (e.g., unjustified angry customer) (Ivanova et al., 2018; Rosenblat and Stark, 2016), or because it is often not transparent which data feeds the performance evaluation of performance (Delfanti, 2019). From an action theoretical perspective, on the one hand this lack of legitimacy and transparency of feedback can impair an important source of learning at work, as unaccepted feedback might be less likely integrated to improve one’s action regulation and on the other hand the action is directed toward less accepted and therefore less intrinsically motivating goals (Christensen-Salem et al., 2018).

**Figure 1:**
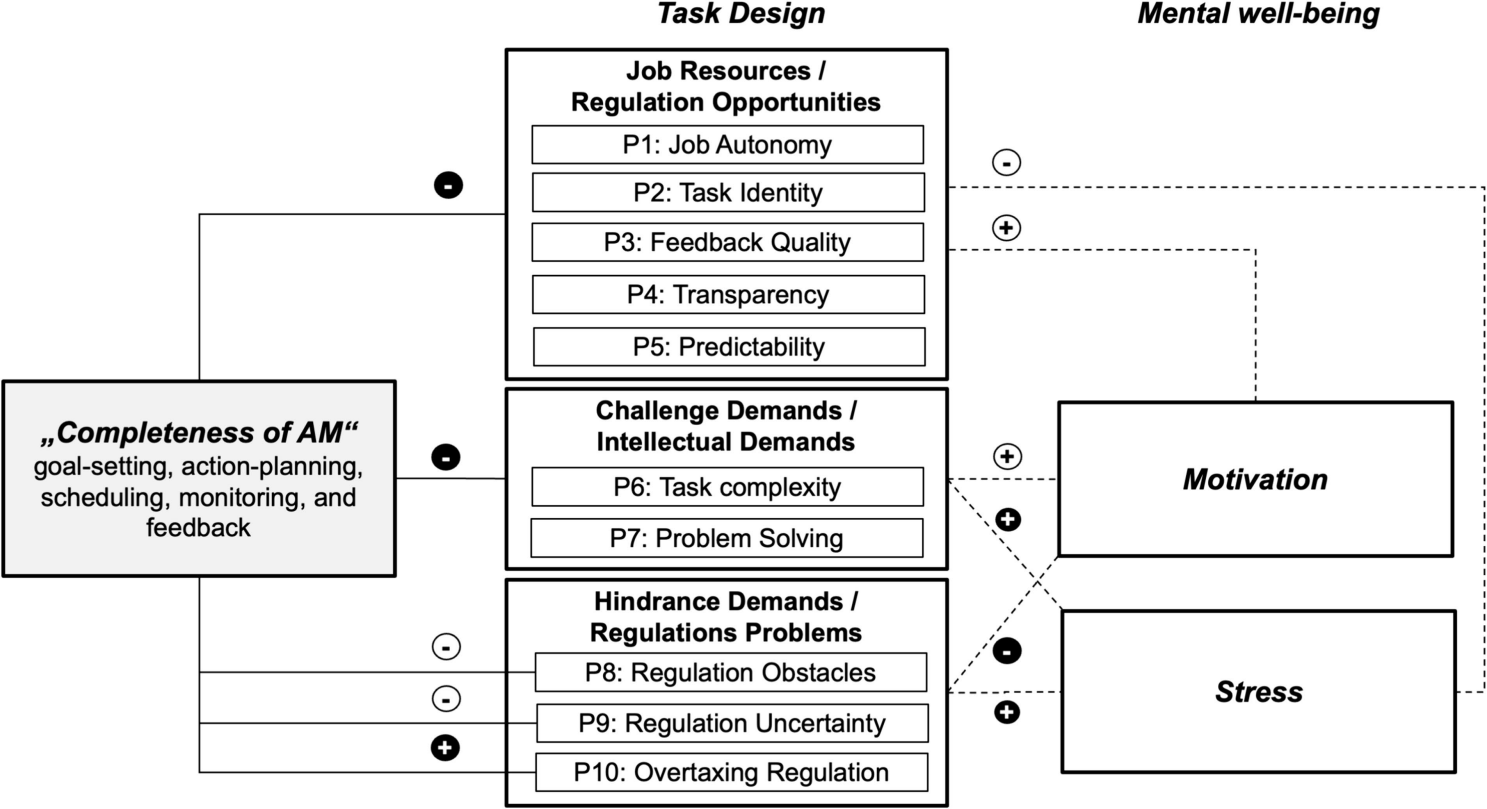
Propositions of associations between completeness of AM and task design. P = Proposition. Black circles indicate detrimental effects on task design according to action regulation theory, as well as on motivation and stress. White circles indicate desirable effects. Dashed lines indicate effects that are not the direct focus of this article but can be derived from the existing literature.

The three use cases indicate that AM as it is predominantly used at present might have similar effects on the completeness of work tasks, independent on the individual work settings. From this, the following propositions can be derived regarding the effects of AM on job resources and job demands, which are important for the regulation of employees’ actions and thus also for their stress experience and motivation. Our propositions are summarized in Figure 1.

Overall we assume that *more complete AM systems are related to reduced job resources* (Parent-Rocheleau and Parker, 2022): As we saw in the use cases, across work settings AM predefines task goals and considerably limits the possibility for workers to influence the goal. Whereas in traditional managed work no constant contact between manager and worker necessitates some form of individual task planning, the close coupling of task assignment and monitoring employs strict rules which hinders autonomy (De Cremer, 2020; Moore and Hayes, 2017). Algorithms are designed in a such a way that make it difficult to intervene in the work process, as human intervention can interfere with the algorithm. Neither the task assignment nor its scheduling is to be easily negotiated or declined. Moreover, especially in the platform economy, workers are often sanctioned when they decline tasks assignment too often (Goods et al., 2019; Gregory, 2021; Griesbach et al., 2019; Lee et al., 2015; Rosenblat and Stark, 2016). This means that AM presumably not only makes it more difficult for workers to make own decisions, but autonomous decisions are also sanctioned in some cases. Finally, as AM systems rely on the quantification of work, workers might focus only on the aspects of work that are reflected in the AM’s quantification of performance. This so called “working for data” has been reported to be related to reduced autonomy (Gal et al., 2020; Schafheitle et al., 2020). Thus, more complete AM should be related to lower *job autonomy* as the central resource for action regulation according to ART (Frese and Zapf, 1994).

*Proposition 1*: More complete AM is associated with lower job autonomy.

We can derive from the above, that AM systems seem to defragment complete tasks. This reduction in task completeness seems to be implied by the need to quantify job demands to enable AM systems to function. Once job demands are fully quantified by an AM system, the optimization and expansion of the system requires that tasks to be repeated as often as possible in the same way. Thus, in logistic warehouses the worker might be only involved in in a small section of the complete task, like taking a product from a shelf or packing it into a box which is then transported to the next step in the delivery process by a conveyer belt. Thus, more complete AM should be related to lower *task identity*.

*Proposition 2*: More complete AM is associated with lower task identity.

Due to the large amount of data available, AM systems have the potential to provide valuable feedback to workers to learn and develop. Unfortunately, research on AM systems implies that this information is rarely shared. Whereas Uber drivers get tips how to improve customer ratings (Rosenblat, 2018; Rosenblat and Stark, 2016), Amazon warehouse workers often not even know on what data their target achievement is based on Delfanti (2019). Moreover, studies report a lack of legitimacy and transparency of feedback criteria, and unreliability of the information source (Ivanova et al., 2018; Rosenblat and Stark, 2016). Thus, although more complete AM should usually provide a large quantity of feedback it should be also related to lower *feedback quality*.

*Proposition 3*: More complete AM is associated with lower feedback quality.

The constant integration of huge amounts of data by AM that is used to adapt action plans and schedules in real time, makes it impossible for the worker to oversee the complete details of a given task before starting to work on it. For example, in the ride sharing business, drivers are only presented with the very next potential passenger pickup address without any details where the customer wants to be taken (Rosenblat and Stark, 2016). The same is true for food delivery services, where a rider needs to accept a task without knowing the delivery address (Griesbach et al., 2019). It is in fact impossible for workers (and managers) to grasp the complete workflow as required information is locked in the AM system (e.g., chaotic storage) (Gent, 2018). Thus, more complete AM should be related to lower *transparency and predictability* of work tasks (Delfanti, 2019; Rosenblat, 2018).

*Proposition 4*: More complete AM is associated with lower transparency.

*Proposition 5*: More complete AM is associated with lower predictability.

We further assume that *more complete AM systems are related to reduced challenge demands:* According to ART *learning* is facilitated by action. With sequentially and hierarchically complete actions workers have the most potential to learn and develop during work (Zacher, 2017). This learning is enabled by both, errors and accomplishments, which the worker integrates via the monitoring and feedback of the action (Frese and Zapf, 1994). In algorithmically managed jobs, however, the work is highly standardized. As already stated above, tasks are broken down into quantifiable steps and often the steps related to one single task are distributed between different workers. Tasks are simplified to a point where workers do not have the opportunity to solve problems by their own (Parent-Rocheleau and Parker, 2022), like being told which box size to use to pack an item or how much tape is required to seal that box (Delfanti, 2019). In addition, the tasks do not vary in complexity. They are merely repetitions of the previous tasks with a different passenger, different food order or different item to pack.

*Proposition 6*: More complete AM is associated with lower task complexity.

*Proposition 7*: More complete AM is associated with lower problem solving.

We finally conclude that *more complete AM has mixed effects on hindrance demands or regulation problems:* In traditionally managed jobs, retrieving required information or getting physically to a desired location or object might pose a challenge. With work being very structured by AM systems with step-by-step guidance, the risk decreases of not having the required information or access rights for the current task. And as there is no choice in which tasks to do next, there is no need to obtain and process such information to structure one’s work (Reyes, 2018). The same seems to be true for the risk of disruptions. In traditionally managed work settings, the unplanned interaction with managers or fellow workers might interrupt the flow of work. With AM workers usually working without any such interactions, a circumstance that represents, however, a work design problem of its own, the main obstacle in an AM workplace would be the malfunction of the AM system.

*Proposition 8*: More complete AM is associated with lower regulation obstacles (i.e., interruptions).

Due to the simplification of work tasks, tasks goals and roles of workers are usually very clearly defined, and role ambiguity should therefore be quite low. Moreover, also regulation uncertainty resulting from the different role expectations of colleagues should be low as workers only very rarely interact at all (Parent-Rocheleau and Parker, 2022). And although the workload is reported to be often high in AM work settings, there is usually a quite low uncertainty on how to achieve a certain goal (Benlian et al., 2022; Cram and Wiener, 2020).

*Proposition 9*: More complete AM is associated with lower regulation uncertainty (i.e., ambiguity or lack of clarity in one’s task goals, or action plans).

*Overtaxing regulations* on the other hand seem to be increased in an AM work environment. AM is reported to increase work demands in terms of workload due to its constant optimization of work efficacy (Gregory, 2021; Reyes, 2018). As performance targets are based on data of past workers AM sets an environment of ever-increasing standards of productivity (Guendelsberger, 2019). For example, in warehouses the daily performance targets are displayed by the scanner and enforced by supervisors (Delfanti, 2019). Due to its reliance on data, AM monitors as many aspects of the task accomplishment as possible and uses this information to control workers behavior with nudging. On several platforms, the algorithmic system modifies pay rates in real time according to demand, thus spurring workers to “chase” lucrative hours, and, consequently, to work long and irregular hours, at times most other people enjoy their free time (Cram and Wiener, 2020; Oppegaard, 2021; Wood et al., 2019). Feedback provided by AM also changes rapidly with the constant updating of algorithms (Stark and Pais, 2020).

*Proposition 10*: More complete AM is associated with higher overtaxing regulation (i.e., time demands, more simultaneous tasks).

## 5 Discussion

The aim of this paper was to complement recent first attempts to establish our conceptual understanding of the psychological impact of algorithmic management (AM) (Gagné et al., 2022; Parent-Rocheleau and Parker, 2022). In line with sociomaterial understandings of technology (Leonardi, 2012) and action regulation theory (ART) (Hacker, 2003; Zacher and Frese, 2018), we propose that the perspective of human action regulation is particularly suited to identify psychologically relevant mechanisms through which AM exerts an effect on workers. From this perspective, we suggest the concept of “completeness” of AM that describes the extent to which AM determines the action steps of a worker, from goals setting to giving feedback. We assume that the degree of “completeness” of AM has a profound effect on the humane design of work tasks, and through task design indirectly also on the motivation and stress experience of workers.

Against this background, we propose that the application of AM can have both negative and positive effects on the human design of work tasks.

On the negative side we expect algorithmically managed tasks to show lower job resources in terms of job autonomy, task identity and feedback quality. The potential to learn and grow is likely to be low as such tasks seem to provide little complexity and opportunities for problem solving. In terms of overtaxing regulation, we expect higher effects due to lower transparency and predictability as well as increased time demands and work intensification. In line with the Challenge-Hindrance Stressor Framework (Podsakoff et al., 2023), we expect some challenge stressors (e.g., complexity) to be limited and some hindrance stressors (e.g., resource inadequacies) to be high under conditions of AM and thus potentially decreasing motivation and increasing stress.

On the positive side, we expect algorithmically managed tasks to show few regulation obstacles and low regulation uncertainty as algorithmically managed tasks follow strict rules and have clearly defined roles and responsibilities. We also expect some challenge stressors (e.g., time pressure) to be high and some hindrance stressors (e.g., role and interpersonal conflict) to be low, having a positive effect on motivation but at the same time also contribute to higher stress. Further positive aspects are assumed to be an increase in flexibility and performance opportunities (Benlian et al., 2022), as well as a positive perception of procedural justice (Bujold and Parent-Rocheleau, 2024).

Thus, in accordance with the Job Demands-Resources Model (Bakker and Demerouti, 2017) we expect a mixed picture of AM on job demands and job resources. As complexity decreases, we expect the tasks to become easier to fulfill while providing less potential for learning. In the same vein, regulation obstacles are expected to be low suggesting a reduction of demotivating and stressful effects. On the other hand, with lower autonomy and less information resources, AM tasks provide less job resources suggesting lower motivational and stress reducing effects as well.

Our paper complements recent psychological conceptualizations of AM within frameworks of work design theory (Parent-Rocheleau and Parker, 2022) and self-determination theory (Gagné et al., 2022). By linking sociomaterial system theory that considers technologies as amalgamation of material and human goals (e.g., Landers and Marin, 2021) with ART (Hacker, 2003; Zacher and Frese, 2018) we show potential underlying cognitive mechanisms in terms of action regulation through which the functions of AM might affect psychosocial important aspects of task design, and with that also workers’ well-being.

Our approach also complements the level of automation (LOA) research (Endsley, 2018) in a meaningful way: From a conceptual perspective, like ours, the LOA approach deals with the psychological effects of different degrees of automation of action steps through the application of technologies. However, LOA focuses specifically on immediate performance-relevant cognitive effects, such as possible impairments of situational awareness, which, among other things, make it more difficult for workers to intervene in critical situations (i.e., out-of-the-loop performance problems). In addition to this, we take a broader perspective by discussing the effects of automation on the design aspects of the work tasks and entire work activity. This perspective allows drawing additional conclusions about long-term motivational and health-related effects of automation which are not in the focus of LOA.

From a more practical perspective, AM can be considered as a specific application of automation that so far is not as much a focus of LOA research. LOA mainly refers to applications were the worker controls the execution of actions by the machine instead of doing the work him- or herself (Endsley, 2018; Vagia et al., 2016). In contrast, in AM machines do not take over action execution from workers to ease or speed up manual work, elevating the workers cognitive efforts (Vagia et al., 2016). Instead, in AM it almost appears to be the opposite split of responsibilities within the human-machine-interaction: workers need to do the manual work machines cannot do yet, losing the cognitive challenging aspects of the task to the AM system.

### 5.1 Limitations of our perspective and recommendations for future research

Our proposed action theoretical approach ignores some perspectives that are also important for the psychological understanding of AM:

Action regulation theory mainly focusses on the individual cognitive processes of actions. It focusses less on the social interactions during these actions as regulation requirements or resources (Zacher and Frese, 2018). According to self-determination theory, social relationships can act as important personal resources leading to the experience of joy and intrinsic motivation as central characteristics of good mental well-being (Deci et al., 2017). Good social relations at work, like social support by managers and colleagues, are one of the main job resources for mental well-being at the workplace (Montano et al., 2017). As the described use cases of AM work environments show mostly solitary tasks at work, the lack of social relationships at work might pose an additional effect on worker well-being (Gagné et al., 2022) that we did not integrate directly into our conceptualization.

Moreover, role-based identity theory (Ramarajan, 2014) suggests that there are work tasks that are perceived as more central to one’s work role than others. Such work tasks are called direct tasks (Gabriel et al., 2011) or core tasks (Semmer et al., 2015). Research indicates that satisfaction of workers with the accomplishment of such core tasks is more strongly related with well-being than satisfaction with the accomplishment of non-core tasks (Gabriel et al., 2011). It seems plausible that the assumed shift of organizational control from workers to technology by the introduction of AM (Kellogg et al., 2020; Möhlmann et al., 2021) might fundamentally change core aspects of work tasks and with that also the workers’ roles. Thus, negative effects of AM can be expected particularly when accepted and internalized work roles are transformed and the work-related identity of employees changes fundamentally (Semmer et al., 2015). This can be particularly expected when AM is introduced in “traditional” jobs, where understanding of one’s own work task has developed across organizations over longer periods of time, in contrast to the new “gig economy,” where such traditional work roles do not yet exist and AM may therefore be more accepted (Baiocco et al., 2022). Although this example is not directly an implementation of AM, in medicine algorithms using artificial intelligence technologies like machine learning have already been proven to be as effective as humans when it comes to diagnosing certain medical conditions. This benefit for patients comes with severe changes for the role of doctors (Loh, 2018). Similar changes occur in human resources management as the activities of sourcing and assessment are more and more handled by algorithms instead of humans (Li et al., 2021; Loh, 2018). One can conclude, that an investigation of AM effects across different professional contexts is needed. In particular, the distinction between jobs in the new “gig economy” and traditional jobs seems appropriate here.

The current body of literature about AM is mainly based on qualitative research (Benlian et al., 2022; Gagné et al., 2022; Parent-Rocheleau and Parker, 2022). Although these studies have provided us with the valuable information this review is built on, quantitative research is required to enable more robust testing of the current hypotheses about the impact of AM on work and task design as well as workers’ well-being. The recently developed algorithmic management questionnaire (Parent-Rocheleau et al., 2023) is one existing instrument starting to be used to quantify the impact of AM (Bujold and Parent-Rocheleau, 2024).

Furthermore, the currently available studies are mostly focusing on very pure (or extreme) applications of AM. Most data were obtained from the platform and gig economy, namely ride-sharing, food delivery or clickworkers. In these work environments, AM often fully replaces human managers and workers are mostly performing tasks requiring low formal education. To gather a more general understanding about AM, we need to see its impact in more traditional work settings as already outlined above.

Moreover, research is needed in settings were AM and human managers coexist: Is there a possible combination of AM and human management which maintains the performance benefits from AM without the supposed negative effects on well-being? One might think of augmented leadership, where machine learning algorithms process employee data to provide leadership suggestions to human managers. Today we already see that more transactional management tasks are carried out by algorithms. It is therefore very probable that at some point empathic and motivational leadership tasks will be supported or even taken over by AM (Quaquebeke and Gerpott, 2023).

### 5.2 Conclusion

There is wide agreement across different academic disciplines that AM will continue to change the way we work (Benlian et al., 2022). The main question is therefore not whether AM is good or bad for workers, but rather *how* work under AM can be designed to be humane. We thus want to stress the importance of work and organizational psychological knowledge in the development of the next generation of AM systems. Today, AM systems are designed and implemented by companies pursuing economic goals. This approach needs to be complemented by scientifically validated design choices reflecting the needs of humans (Parker and Grote, 2022). While some of the negative effects described above should be attributed to inherent and hardly changeable functions of AM systems (such as the restrictions on job autonomy, or the reduction of task identity), other negative effects of AM probably derive not from the technology itself, but from the design choices made by human developers. Managers and developers of AM systems should be informed about potential consequences of design choices for worker well-being. Our considerations suggest that more complete AM systems may be associated with detrimental task design and therefore with risks to the well-being of workers. The technical possibilities of AM systems should therefore not be fully exploited. Instead, human capabilities und needs should be the starting point for the design of AM systems. From an action regulation perspective, AM systems should be used to specifically increase regulation opportunities for workers and eliminate regulation problems. AM systems could provide workers with information they need to make informed and autonomous decisions for themselves and not dictate the decisions to the workers. For example, it is possible to give an Uber driver more information about the location of a potential passenger and not to sanction refusals of ride requests. In this way, AM systems can also minimize obstacles and uncertainties at work. Both could increase the workers’ sense of autonomy and control over their work, and with that also their motivation and mental well-being. Such a human-centered design of AM systems makes it necessary that workers are continuously involved in the development of AM systems, including decisions about the data to be processed and the corresponding algorithms. From the perspective taken here, both example measures should contribute to higher motivation and lower stress levels among workers. The common goal of industry and academia needs to be the improvement of AM as socio-technical systems consisting of both, algorithms and humans, making use of and reflecting their capabilities and needs. In sum, against the background of the above-described state of psychological research on AM, future research should hence advance and systematize the psychological understanding of AM and its functions across different jobs and industries. The here proposed action-theoretical perspective might be a starting point for developing further hypotheses and explaining phenomena.

## Data Availability

The original contributions presented in the study are included in the article/supplementary material, further inquiries can be directed to the corresponding author.
